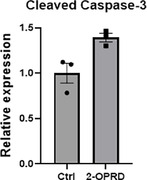# Pathogenic Mechanism of PRNP 2‐Octapeptide Deletion Mutation in Creutzfeldt‐Jakob Disease

**DOI:** 10.1002/alz.090632

**Published:** 2025-01-03

**Authors:** Yiming Wu, Haitian Nan, Yan Zhang

**Affiliations:** ^1^ The Experimental High School Attached to Beijing Normal University, Beijing, Beijing China; ^2^ Xuanwu Hospital, Capital Medical University, Beijing China; ^3^ Peking University, Beijing, Beijing China

## Abstract

**Background:**

Prion diseases are a group of neurodegenerative diseases associated with prion protein. The disease can be caused by mutations in the *PRNP* gene, the gene that encodes prion protein. An octapeptide repeat on the N‐terminus of prion protein plays an important role in normal intercellular function of prion protein. At present, numerous prion disease patients with *PRNP* 2‐octapeptide deletion mutation case have been reported, but it is still unclear whether and how the mutation causes the disease. In this study, we aim to preliminarily identify the pathogenicity of *PRNP* 2‐octapeptide deletion mutation.

**Method:**

The gDNA sample was obtained from a patient with familial creutzfeldt‐Jakob disease. The gDNA was identified to contain heterozygous *PRNP* 2‐octapeptide mutation deletion. We separated and amplified the gene and constructed N1 plasmid models with overexpression of the gene. Finally, we transfected HEK‐293T cell with the plasmid and used Western Blot to quantify the difference of apoptosis level of the mutated gene group and the control group.

**Result:**

Due to limited sample size, the result shows no significant statistical difference between the experimental group and the control group, but still reflected potential trend of increased apoptosis (p<0.1).

**Conclusion:**

Further study of this mutant gene is necessary to determine its pathogenicity.